# PER1 rs3027172 Genotype Interacts with Early Life Stress to Predict Problematic Alcohol Use, but Not Reward-Related Ventral Striatum Activity

**DOI:** 10.3389/fpsyg.2016.00464

**Published:** 2016-03-31

**Authors:** David A. A. Baranger, Chloé Ifrah, Aric A. Prather, Caitlin E. Carey, Nadia S. Corral-Frías, Emily Drabant Conley, Ahmad R. Hariri, Ryan Bogdan

**Affiliations:** ^1^Brain Laboratory, Department of Psychological and Brain Sciences, Washington University in St. LouisSt. Louis, MO, USA; ^2^Division of Biology and Biomedical Sciences, Washington University in St. LouisSt. Louis, MO, USA; ^3^Department of Psychiatry, University of California, San FranciscoSan Francisco, CA, USA; ^4^Department of Psychiatry, Washington University in St. LouisSt. Louis, MO, USA; ^5^23andMeMountain View, CA, USA; ^6^Laboratory of NeuroGenetics, Department of Psychology and Neuroscience, Duke UniversityDurham, NC, USA

**Keywords:** circadian, alcohol, stress, PER1, ventral striatum, GxE, early life stress

## Abstract

Increasing evidence suggests that the circadian and stress regulatory systems contribute to alcohol use disorder (AUD) risk, which may partially arise through effects on reward-related neural function. The C allele of the *PER1* rs3027172 single nucleotide polymorphism (SNP) reduces *PER1* expression in cells incubated with cortisol and has been associated with increased risk for adult AUD and problematic drinking among adolescents exposed to high levels of familial psychosocial adversity. Using data from undergraduate students who completed the ongoing Duke Neurogenetics Study (DNS) (*n* = 665), we tested whether exposure to early life stress (ELS; Childhood Trauma Questionnaire) moderates the association between rs3027172 genotype and later problematic alcohol use (Alcohol Use Disorders Identification Test) as well as ventral striatum (VS) reactivity to reward (card-guessing task while functional magnetic resonance imaging data were acquired). Initial analyses found that *PER1* rs3027172 genotype interacted with ELS to predict both problematic drinking and VS reactivity; minor C allele carriers, who were also exposed to elevated ELS reported greater problematic drinking and exhibited greater ventral striatum reactivity to reward-related stimuli. When gene × covariate and environment × covariate interactions were controlled for, the interaction predicting problematic alcohol use remained significant (*p* < 0.05, corrected) while the interaction predicting VS reactivity was no longer significant. These results extend our understanding of relationships between *PER1* genotype, ELS, and problematic alcohol use, and serve as a cautionary tale on the importance of controlling for potential confounders in studies of moderation including gene × environment interactions.

## Introduction

Observable psychiatric symptoms (e.g., insomnia/hypersomnia) and biological rhythm perturbation (e.g., dysregulated diurnal cortisol) have been linked to variability in circadian rhythm function (Nader et al., 2009; Wulff et al., 2009; Chong et al., 2012; Wirth et al., 2013). Accumulating cross-species evidence highlights a bidirectional relationship between the circadian system and alcohol consumption; circadian manipulations induce changes in alcohol consumption while alcohol intake impacts circadian rhythm-related gene expression (Spanagel et al., 2005; Kovanen et al., 2010; Gamsby et al., 2013; McCarthy et al., 2013). Further evidence suggests that stress, one of the most potent provocateurs of alcohol use (Enoch, 2011), may play an important role in links between alcohol use and circadian rhythm dysregulation, through interactions with the stress-regulatory neuroendocrine hypothalamic pituitary adrenal (HPA) axis (Sarkar, 2012).

The circadian system is governed by a system of transcriptional repressors (i.e., Period genes: *PER1, PER2, PER3*; Cryptochrome genes: *CRY1, CRY2*) and enhancers (i.e., *CLOCK* and *BMAL1*) that influence numerous downstream clock-responsive genes to maintain a 24-h biochemical (e.g., hormone production), physiological (e.g., brain function, body temperature), and behavioral (e.g., sleep, eating) cycle (Sarkar, 2012). The maintenance of this daily oscillation is disrupted by stress (Meerlo et al., 2002) with intriguing evidence that mutual interactions among the circadian system and HPA axis may mediate these effects (Nader et al., 2010) and importantly contribute to problematic alcohol use (Dong et al., 2011; Blomeyer et al., 2013). The period 1 gene (*PER1*) plays a prominent role integrating the circadian system and HPA axis, with recent evidence that it may be critical for understanding problematic drinking behavior. *mPer1* null mutant mice (*mPer1*^*Brdm1*^) have increased ethanol intake and conditioned place preference (Gamsby et al., 2013). Moreover, highlighting the potential etiologic role of stress and the HPA axis in this relationship, these mice display stress-induced (social defeat, swim stress, or foot shock) increases in ethanol consumption (Dong et al., 2011) and impaired glucocorticoid rhythmicity (Dallmann et al., 2006).

While the specific mechanisms by which stress and circadian disruption modulate alcohol consumption remain to be elucidated, evidence suggests that altered neural processing of rewards may play a mediating role. Individuals with alcohol use disorders (AUD) and those at genetic risk for their development have differential ventral striatum responses to non-alcohol rewards (Beck et al., 2009; Yau et al., 2012). Further, sleep deprivation is associated with enhanced striatal reactivity to rewards in humans (Venkatraman et al., 2011; Mullin et al., 2013). Moreover, early life stress (ELS) is associated with reduced D2 dopamine receptor positive cells in the striatum of rodents (Li et al., 2013) and reduced ventral striatal activation to rewards in human participants (Dillon et al., 2009; Boecker et al., 2014).

In humans, a single nucleotide polymorphism (SNP) within *PER1*, rs3027172, has been associated with individual differences in cortisol-dependent gene expression as well as problematic drinking in the context of environmental adversity (Dong et al., 2011). Specifically, the minor C allele at rs3027172, which leads to reduced *PER1* expression in B-lymphoblastoid cell lines incubated with cortisol, predicts elevated rates of alcohol dependence among adults and problematic drinking among adolescents exposed to prenatal familial psychosocial adversity. Using data from the ongoing Duke Neurogenetics Study (DNS) (*n* = 665), which assesses a wide range of behavioral, experiential, and biological phenotypes in university students, the present study examined whether *PER1* rs3027172 genotype and ELS interact to predict problematic alcohol use. Moreover, given evidence that *PER1* influences response to reward (Abarca et al., 2002; Liu et al., 2007) and that individuals with AUD or those at genetic risk for their development have differential ventral striatum response to non-alcohol reward (Beck et al., 2009; Yau et al., 2012), we further examined whether *PER1* rs3027172 genotype and ELS predict variability in reward-related ventral striatum reactivity, which may play a mediating role linking *PER1* rs3027172 genotype and ELS to problematic alcohol use.

## Materials and methods

### Participants

Overlapping neuroimaging and genetic data that were fully processed by January 6th 2014 were available from 727 participants who completed the DNS. The DNS assesses a wide range of behavioral, experiential, and biological phenotypes among young-adult (i.e., 18–22 year-old) college students. Each participant provided informed written consent prior to participation in accord with the Declaration of Helsinki and guidelines of the Duke University Medical Center Institutional Review Board. Participants received $120 remuneration. All participants were in good general health and free of DNS exclusion criteria: (1) medical diagnosis of cancer, stroke, diabetes requiring insulin treatment, chronic kidney or liver disease or lifetime psychotic symptoms; (2) use of psychotropic, glucocorticoid or hypolipidemic medication, and (3) conditions affecting cerebral blood flow and metabolism (e.g., hypertension). Current DSM-IV Axis I and select Axis II disorders (Antisocial Personality Disorder and Borderline Personality Disorder) were assessed with the electronic Mini International Neuropsychiatric Interview (Sheehan et al., 1998) and Structured Clinical Interview for the DSM-IV Axis II (SCID-II) (First et al., 1997). These disorders are not exclusionary as the DNS seeks to establish broad variability in multiple behavioral phenotypes related to psychopathology.

The final sample consisted of 665 participants after quality assurance (age = 19.64 ± 1.24; 294 males; 123 with a DSM-IV Axis I disorder; 305 European Americans, 73 African Americans, 187 Asians, 39 Latinos, and 61 of Other/Multiple racial origins according to self-reported ethnicity). Participants were excluded (*n* = 62) for scanner-related artifacts in fMRI data (*n* = 5), incidental structural brain abnormalities (*n* = 2), a large number of movement outliers in fMRI data (*n* = 31; see ART below), poor behavioral performance or an inadequate feedback schedule (*n* = 11), outlier status according to ancestrally-informative principal components (*n* = 6), scanner malfunction (*n* = 2), incomplete fMRI data collection (*n* = 1), missing or uncollected task behavioral data (*n* = 1), and subjects falling asleep (*n* = 2). An additional participant was excluded as they did not complete the questionnaires used for these analyses (*n* = 1). Comparison of participants excluded due to lack of neuroimaging data to those included found no significant differences (Supplemental Table 1).

### Self-report questionnaires

Participants completed a battery of self-report questionnaires to assess past and current experiences and behavior. The Childhood Trauma Questionnaire (CTQ; Bernstein et al., 2003), the Alcohol Use Disorders Identification Test (AUDIT; Saunders et al., 1993), and the Pittsburgh Sleep Quality Inventory (PSQI; Buysse et al., 1989) were used for the present study. The CTQ is a 28-item, retrospective screening tool used to detect the occurrence and frequency of emotional, physical, and sexual abuse as well as emotional and physical neglect before the age of 17. The instrument's five subscales, each representing one type of abuse or neglect, have robust internal consistency and convergent validity with a clinician-rated interviews of childhood abuse (Scher et al., 2001). Each subscale with the exception of physical neglect had adequate internal consistency (Emotional Abuse α = 0.765, Physical Abuse α = 0.677, Sexual Abuse α = 0.886, Emotional Neglect α = 0.849, Physical Neglect α = 0.422). As our goal was to capture overall ELS, we used the CTQ total for analyses, which had an α of 0.654. This is slightly below standard levels of 0.70, likely due to the heterogeneity of ELS assessed. Notably, these α values are comparable to those observed in prior studies (Scher et al., 2001). The AUDIT is a 10-item scale developed by the World Health Organization to screen for hazardous or dependent alcohol use patterns by assessing the frequency and nature of consumption (α = 0.799); a score of 8 or greater is considered indicative of hazardous or harmful use (Saunders et al., 1993). While the AUDIT was originally developed to screen for alcohol use problems and high-risk drinking in primary care settings, evidence suggests that it is a valid assessment for college student populations as well (Kokotailo et al., 2004). The PSQI is a 19-item scale that is widely used and considered a reliable measure of global sleep quality and sleep-related symptoms over the past 1 month (α = 0.727). Scores range from 0 to 21, with poorer sleep quality associated with a higher score.

### Genotyping

DNA was isolated from saliva derived from Oragene DNA self-collection kits (DNA Genotek) customized for 23andMe (www.23andme.com). DNA extraction and genotyping were performed through 23andMe by the National Genetics Institute (NGI), a CLIA-certified clinical laboratory and subsidiary of Laboratory Corporation of America. One of two different Illumina arrays with custom content was used to provide genome-wide SNP data, the HumanOmniExpress or HumanOmniExpress-24 (Eriksson et al., 2010; Do et al., 2011; Tung et al., 2011; Hu et al., 2016).

*PER1* rs3027172 was directly genotyped for 324 participants. It was imputed for the remaining 403. Imputation was run separately for participants genotyped on the Illumina HumanOmniExpress and the Illumina HumanOmniExpress-24 arrays using biallelic SNPs only, the default value for effective size of the population (20,000), and chunk sizes of 3 Mb and 5 Mb for the respective arrays. Within each array batch, genotyped SNPs used for imputation were required to have missingness < 0.02, Hardy-Weinberg equilibrium *P* > 10^−6^, and MAF > 0.01. The imputation reference set consisted of 2504 phased haplotypes from the full 1000 Genomes Project Phase 3 dataset (May 2013, over 70 million variants, release “v5a”). Imputed SNPs were retained if they had high imputation quality (INFO > 0.9), low missingness (< 5%), and MAF > 0.01. *PER1* rs3027172 had excellent imputation metrics (INFO = 0.997, Certainty = 0.999). Genotype frequencies did not deviate from Hardy-Weinberg Equilibrium across any ancestral group (HWE: χ^2^ = 1.97, *p* = 0.85; HWE Caucasian: χ^2^ = 1.03, *p* = 0.78; African-American: 0.96, *p* = 0.90; Asian1: χ^2^ = 0.24, *p* = 0.93; Asian2: χ^2^ = 0.38, *p* = 0.95; Hispanic: χ^2^ = 0.65, *p* = 0.82; Other χ^2^ = 1.83, *p* = 0.85).

To account for differences in ancestral background in the full sample, we used EIGENSTRAT (v. 5.0.1) (Price et al., 2006) to generate principal components; k-means cluster plotting and visual inspection of the top 10 components revealed that the top 5 principal components account for divergent ancestral groups within the population (Supplemental Figure 1). Six participants were identified as outliers, as they were more than 6 standard deviations from the mean on these top 5 components, and were excluded from analyses. Ancestral subsamples were determined based on self-report (Caucasian, African American, Hispanic, Asian, or Other), except in the case of the Asian sample, which, based on visual inspection of the principal components produced by EIGENSTRAT, was composed of two genetically distinct subsamples. Self-reported Asians were thus further divided into two subsamples (Asian1: *n* = 47; Asian2: *n* = 147) based on k-means clustering of the first two principal components.

### BOLD fMRI paradigm

A number guessing paradigm (Delgado et al., 2000) was used to probe reward-related VS activity. Our blocked design consisted of a pseudorandom presentation of three blocks each of predominantly positive (80% correct guess) and negative (20% correct guess) feedback. There are five trials during each block. During each task trial, subjects had 3 s to guess, via button press, whether the value of an upcoming visually presented card would be < or >5 (index and middle finger, respectively). The numerical value of the card was presented for 500 ms followed by appropriate feedback (i.e., green “up” arrow for positive feedback on a correct trial; red “down” arrow for negative feedback on an incorrect trial) for an additional 500 ms. A crosshair focus point was then presented for 3 s for a total trial length of 7 s. One incongruent trial type was included within each task block to prevent subjects from anticipating the feedback for each trial and maintain subject's engagement and motivation to perform well. The six task blocks were interleaved with three control blocks. During control blocks, subjects were instructed to make button presses during the presentation of an “x” (3 s), which was followed by an asterisk (500 ms) and a yellow circle (500 ms). Each block was preceded by a 2 s instruction of “Guess Number” (for task) or “Press button” (for control), resulting in a total block length of 38 s and a total task length of 342 s. Subjects were unaware of the fixed outcome probabilities associated with each block and were led to believe that their performance would determine their net monetary gain, although all subjects received $10 upon completion of the task.

### BOLD fMRI acquisition

Participants were scanned using a research-dedicated GE MR750 3T scanner equipped with high-power high-duty-cycle 50-mT/m gradients at 200 T/m/s slew rate, and an eight-channel head coil for parallel imaging at high bandwidth up to 1 MHz at the Duke-UNC Brain Imaging and Analysis Center. A semi-automated high-order shimming program was used to ensure global field homogeneity. A series of 34 interleaved axial functional slices aligned with the anterior commissure-posterior commissure (AC-PC) plane were acquired for full-brain coverage using an inverse-spiral pulse sequence to reduce susceptibility artifact [TR/TE/flip angle = 2000 ms/30 ms/60; FOV = 240 mm; 3.75 × 3.75 × 4 mm voxels (selected to provide whole brain coverage while maintaining adequate signal-to-noise and optimizing acquisition times); interslice skip = 0]. Four initial RF excitations were performed (and discarded) to achieve steady-state equilibrium. To allow for spatial registration of each participant's data to a standard coordinate system, high-resolution three-dimensional structural images were acquired in 34 axial slices co-planar with the functional scans (TR/TE/flip angle = 7.7 s/3.0 ms/12; voxel size = 0.9 × 0.9 × 4 mm; FOV = 240 mm, interslice skip = 0).

### BOLD fMRI data analysis

The general linear model of Statistical Parametric Mapping 8 (SPM8) (http://www.fil.ion.ucl.ac.uk/spm) was used for whole-brain image analysis. Individual subject data were first realigned to the first volume in the time series to correct for head motion before being spatially normalized into the standard stereotactic space of the Montreal Neurological Institute (MNI) template using a 12-parameter affine model. Next, data were smoothed to minimize noise and residual differences in individual anatomy with a 6 mm FWHM Gaussian filter. Voxel-wise signal intensities were ratio normalized to the whole-brain global mean. Then the ARTifact Detection Tool (ART) (http://www.nitrc.org/projects/artifact_detect/) was used to generate regressors accounting for images due to large motion (i.e., >0.6 mm relative to the previous time frame) or spikes (i.e., global mean intensity 2.5 standard deviations from the entire time series). Participants for whom more than 5% of acquisition volumes were flagged by ART (*n* = 30) were removed from analyses. A 5 mm sphere based on the maximum voxels from Hariri et al. (2006) was used to ensure adequate ventral striatal coverage; no subjects had < 90% coverage of the region.

Following preprocessing steps outlined above, linear contrasts employing canonical hemodynamic response functions were used to estimate task-specific BOLD responses for each individual using a “Positive Feedback > Negative Feedback” contrast. Individual contrast images (i.e., weighted sum of the beta images) were used in second-level random effects models accounting for scan-to-scan and participant-to-participant variability to determine mean contrast-specific responses using one-sample *t*-tests. A voxel-level statistical threshold of *P* < 0.05, family wise error corrected for multiple comparisons across the bilateral ventral striatal region of interest (ROI), and a cluster-level extent threshold of 10 contiguous voxels was applied to these analyses. The bilateral ventral striatal ROI was defined by a 5 mm sphere based on the maximum voxels from Hariri et al. (2006), created with the Wake Forest University PickAtlas (Lancaster et al., 2000; Maldjian et al., 2003) (Supplemental Figure 2).

BOLD parameter estimates from clusters within the left and right ventral striatal ROIs exhibiting a main effect for the “Positive Feedback > Negative Feedback” contrast were extracted using the VOI tool in SPM8 (http://www.fil.ion.ucl.ac.uk/spm) and exported for regression analyses. Bilateral ROI values were calculated by weighting mean activity in each hemisphere by cluster size and then averaging across the hemispheres. Extracting parameter estimates from clusters activated by our fMRI paradigm, rather than those specifically correlated with our independent variables of interest, precludes the possibility of any correlation coefficient inflation that may result when an explanatory covariate is used to select a ROI. We have successfully used this strategy in prior studies (Carré et al., 2012; Corral-Frías et al., 2016).

### Statistical analyses

Extracted neuroimaging data values were winsorized (to ±3 SDs; *n* = 11) to maintain variability while limiting the influence of extreme outliers before being analyzed in PASW Statistics (Version 19; SPSS Inc.; Chicago, IL). A regression-based moderation model was tested using the PROCESS macro for SPSS (Hayes, 2013) to examine the independent and interactive effects of ELS (i.e., CTQ score) and *PER1* rs3027172 genotype on problematic alcohol use (i.e., AUDIT score) and reward-related ventral striatum reactivity (i.e., positive reward > negative loss). CTQ scores were log-transformed for all analyses, as they had a high positive skew (Supplemental Table 2). As there were only 20 *PER1* rs3027172 minor allele (C) homozygotes in the sample (3.0%), and 162 *PER1* rs3027172 heterozygotes, *PER1* genotype was coded as the presence or absence of the minor-allele, consistent with prior studies (Dong et al., 2011). A power analysis conducted with Quanto (v.1.2.4) using the effect size previously observed by Dong and colleagues, and our observed genotype frequency and CTQ distribution, revealed that the current sample has 80% power to detect GxE interaction effects greater than β = 0.119 (Gauderman, 2002a,b). Initial moderation analyses were conducted using sex, age (i.e., above or under 21; the legal drinking age in North Carolina), sleep quality (PSQI score), the presence of a psychiatric diagnosis, and the top 5 principal components accounting for divergent ancestral groups within the population (Supplemental Figure 1). Sleep quality was included as a covariate as sleep disruption is associated with increased risk for drug problems (Wong et al., 2010), and variants within other circadian genes have been associated with sleep phenotypes (Hu et al., 2016). Controlling for sleep quality thus permits examination of the effects of PER1 rs3027172 independent of any potential associations of sleep quality. Consistent with recommendations (Keller, 2014), additional follow-up moderation analyses included 18 additional terms for gene × covariate and environment × covariate interactions to better account for potential confounds to GxE research (e.g., *PER1* rs3027172 × sex, etc.; Keller, 2014). Thus, two *a priori* analyses were conducted, yielding a bonferroni correction significance threshold of *p* < 0.025. Given the ethnic diversity of the sample, *post-hoc* analyses in each of the six ancestral subsamples were conducted with recalculated covariate interaction terms. All covariates were the same as in the full-sample analyses, with the exception of the ancestral principal components, which were not included. Additionally, as only 23.6% of the sample had an AUDIT score of 8 or more, which qualifies as hazardous use of alcohol, an additional *post-hoc* logistic regression analysis was conducted in the full-sample to examine whether the interaction of *PER1* rs3027172 and CTQ also predicts the likelihood of an AUDIT score of 8 or more, indicative of more severe problematic drinking.

## Results

### Associations with sample demographics

Consistent with prior observations, men reported more problematic alcohol use (Hasin et al., 2007) and had higher bilateral reward-related VS reactivity to monetary gains (Spreckelmeyer et al., 2009; Nikolova et al., 2012; Supplemental Table 3). Ethnicity predicted self-report measures of stress, sleep, and alcohol use (Supplemental Table 4). Notably, African American and Asian 2 participants were characterized by relatively greater CTQ scores and reduced AUDIT scores, while Caucasian participants reported reduced CTQ scores and elevated AUDIT scores. African American participants also reported higher PSQI scores. *PER1* rs3027172 genotype groups differed by ethnicity, wherein the minor allele carrier group had a higher percentage of Caucasian and a lower percentage of Asian1 and Asian2 participants (Supplemental Table 5; Supplemental Figure 1). Consistent with a prior report (Dong et al., 2011), *PER1* rs3027172 genotype groups differed according to AUDIT scores such that C allele carriers reported higher levels of problematic drinking (Supplemental Table 5); notably, however, this effect did not remain after controlling for covariates (see below). *PER1* rs3027172 genotype groups did not differ by CTQ scores, suggesting the lack of rGE.

### PER1 rs3027172 and early life stress interact to predict problematic drinking

**Figure 1:**
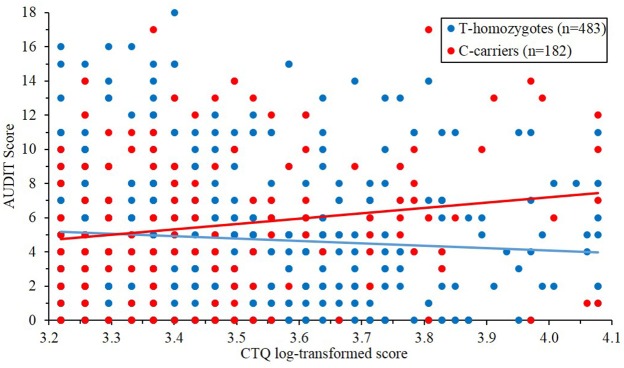
***PER1* rs3027172 and early life adversity interact to predict problematic drinking (Δ*R*^2^ = 0.0106, β = 0.124, *t* = 2.86, *p* = 0.00438)**. C minor-allele carriers report increased problematic drinking behavior (AUDIT scores) in the context of early life stress (CTQ scores). The purple-shaded region denotes the regions of significance (i.e., CTQ log-transformed >3.57, equivalent to a score of 35.5).

There was no main effect of *PER1* genotype or CTQ scores on AUDIT scores after accounting for covariates (*PER1:* β = 0.025, *t* = 0.662, *p* = 0.508; CTQ: β = −0.039, *t* = −0.983, *p* = 0.325; Supplemental Table 5). Initial moderation analyses found that the interaction of *PER1* with ELS (CTQ scores) significantly predicted problematic drinking (Δ*R*^2^ = 0.0067, β = 0.086, *t* = 2.275, *p* = 0.023) after accounting for main effects and covariates. This interaction remained significant after accounting for 2-way interactions between covariates with *PER1* rs3027172 and CTQ scores (an additional 18 covariates; Δ*R*^2^ = 0.0106, β = 0.124, *t* = 2.86, *p* = 0.004; Supplemental Table 6). *Post-hoc* analyses revealed that minor (C) allele carriers who retrospectively reported elevated ELS (Johnson-Neyman significance for log-transformed CTQ values >3.57, = 35.5) endorsed increased problematic drinking (Figure 1). Participants were partitioned into three groups based on the distribution of CTQ-scores (low = 3.22–3.37; medium = 3.37–3.59; high = 3.59–4.08) for *post-hoc* examination of simple slopes. These analyses revealed that *PER1* rs3027172 was associated with increased problematic drinking only in the high CTQ group (β = 1.908, *t* = 2.474, *p* = 0.014). These results are consistent with prior reports of increased heavy drinking among adolescent *PER1* rs3027172 minor-allele carriers who have experienced high levels of psychosocial adversity (Dong et al., 2011). We further examined whether the *PER1* × CTQ interaction predicted the likelihood of an AUDIT score over 8 (defined as the threshold for hazardous use). Logistic regression revealed that the *PER1* × CTQ interaction was significantly associated with this AUDIT threshold of hazardous use (Δ*R*^2^ = 0.0087, β = 0.5908, *z* = 2.128, *p* = 0.033; Supplemental Table 7).

Given the ethnic diversity of the sample, *post-hoc* analyses were conducted in each ancestral sub-sample (Supplemental Table 8, Supplemental Figure 3). In these analyses the interaction of *PER1* and CTQ scores predicting AUDIT scores was only significant in one of the six subsamples (Asian 1; Δ*R*^2^ = 0.153, β = 0.618, *t* = 3.4560, *p* = 0.002), which was notably small (*n* = 38 major allele homozygotes, 6 minor allele carriers). However, in five subsamples (Caucasian, African American, Asian 1, Asian 2, Other) the interaction coefficient was also positive and the shape of the interaction resembled the results from the entire sample across all subsamples (Supplemental Table 8; Supplemental Figure 3). Finally, the original association of *PER1* × CTQ with AUDIT scores was repeated including participants originally excluded due to lack of imaging data (*n* = 719); results did not meaningfully change (Δ*R*^2^ = 0.0119 β = 0.129, *t* = 3.18, *p* = 0.002; Supplemental Table 9).

### PER1 rs3027172 and early life stress do not interact to predict ventral striatal reactivity

Initial moderation analyses found that *PER1* rs3027172 genotype interacted significantly with ELS (CTQ scores) to predict bilateral ventral striatal reactivity (Δ*R*^2^ = 0.0068, β = 0.0838, *t* = 2.145, *p* = 0.032). In the context of high ELS, minor-allele carriers had elevated ventral striatal reactivity. However, this interaction became non-significant after accounting for gene (*PER1* rs3027172) × covariate and environment (CTQ) × covariate interactions (Δ*R*^2^ = 0.0021, β = 0.056, *t* = 1.231, *p* = 0.219; Supplemental Figure 4, Supplemental Table 10). *Post-hoc* analyses indicated that the CTQ × genotype interaction was no longer significant after the inclusion of the CTQ × ancestral principal component 1 (PC1) interaction term (β = −12.3664, *t* = −2.51, *p* = 0.012; Supplemental Figure 5), and the CTQ × PSQI interaction term (β = −0.07234, *t* = −3.114, *p* = 0.002; Supplemental Figure 6). PC1 correlates with PER1 genotype (Pearson's *r* = −0.237, *p* < 0.001 and membership to the White, African American, Asian 2, and Hispanic subgroups; White: Pearson's *r* = −0.625, *p* < 0.001, African American: Pearson's *r* = −0.177, *p* < 0.001; Asian 2: Pearson's *r* = 0.951, *p* < 0.001, Hispanic: *r* = −0.099, *p* < 0.05), and the CTQ × PC1 interaction term correlates with membership to the African American and Asian2 subgroups (African American: Pearson's *r* = −0.150, *p* < 0.001; Asian2: *r* = 0.228, *p* < 0.001). This suggests that analyses that did not account for gene × covariate and environment × covariate interactions, were confounded by an interaction between ancestral origin and ELS. Lastly, given the ethnic diversity of the sample, *post-hoc* analyses were conducted in each ancestral sub-sample (Supplemental Table 11). In these analyses the interaction of *PER1* and CTQ scores predicting ventral striatal reactivity was only significant in one of the six subsamples (African American; Δ*R*^2^ = 0.0556, β = −0.31384, *t* = −2.082, *p* = 0.042). Notably the direction of this interaction is negative, while the coefficient in the full sample is positive. However, in the five other subsamples the coefficient (i.e., positive) and shape of the interaction was in the same direction as in the full-sample analysis. Because the *PER1* × CTQ interaction was not associated with individual differences in reward-related ventral striatum activity when accounting for gene × covariate and environment × covariate interactions we did not test a moderated mediation model.

## Discussion

This study examined whether the *PER1* SNP, rs3027172, interacts with ELS to predict problematic alcohol use and ventral striatum reactivity to reward. Two primary findings emerged. First, consistent with past research (Dong et al., 2011), minor C allele carriers who were exposed to elevated levels of childhood stress, had higher problematic alcohol use (Figure 1). Second, in contrast to initial analyses suggesting that this interaction also predicts reward-related ventral striatum reactivity, when we appropriately accounted for gene × covariate and environment × covariate interactions (Keller, 2014), this interaction was no longer significant. Collectively, these findings provide additional evidence that psychosocial adversity during childhood confers risk for problematic drinking in rs3027172 C allele carriers, but suggest that this association is not driven, at least primarily, by effects on reward-related ventral striatum reactivity. More broadly, these findings highlight the need to account for gene × covariate and environment × covariate interactions in gene × environment and other forms of moderation-based research (Keller, 2014).

### *PER1* rs3027172 genotype and early life stress interact to predict problematic alcohol use

Consistent with a prior report showing that *mPer1*^*Brdm1*^ knockout mice and human minor C allele carriers at rs3027172 have increased alcohol consumption in the context of prenatal adversity (Dong et al., 2011), we found that young-adult C allele carriers had increased problematic alcohol use in the context of elevated ELS. Notably, while Dong et al. (2011) evaluated psychosocial adversity within the family during the year prior to birth, ELS was evaluated in the present study as stress experienced during childhood. However, contrary to Dong et al. (2011), who observed a main effect of *PER1* genotype on risk for alcohol abuse in their second sample, consisting of 2184 Caucasian adults, we did not find any significant main-effects of *PER1* rs3027172 after accounting for covariates (notably, this main effect was significant and in the direction reported by Dong et al. (2011) when covariates were not included; Supplemental Table 5). It is possible that we did not observe such a main effect due to our younger aged sample, the smaller sample size, and ethnic heterogeneity.

Given that *PER1* expression is sensitive to stress, it is not entirely surprising that the minor C allele was only associated with increased problematic alcohol use in the context of ELS. *mPer1* expression in rodents is upregulated in peripheral tissues by acute stress (Yamamoto et al., 2005), and downregulated in the nucleus accumbens by chronic stress (Spencer et al., 2013). Accumulating evidence suggests these stress effects may be mediated through the HPA axis. In human and rodent cell cultures, *PER1* is upregulated by dexamethasone, a glucocorticoid receptor agonist (Reddy et al., 2009; Polman et al., 2012) with evidence that *PER1* is the most sensitive, of all genes, to low doses of dexamethasone (Reddy et al., 2012). Moreover, rs3027172 is located in the *PER1* promoter in a region that is similar to an E2-box binding site for members of the Snail transcription factor family. Snail transcription factors are well-known for their central role in mesoderm formation (Nieto, 2002), are expressed throughout the adult brain (Dong et al., 2011), and have been repeatedly shown to be regulated by stress hormones (for recent examples see Cheng et al., 2013; Nesan and Vijayan, 2013; Shan et al., 2014). The minor C allele, which eliminates the similarity of this site to an E2-box, appears to reduce affinity of Snail1 for this binding site, and results in a four-fold reduction of *PER1* mRNA expression in B-lymphoblastoid cell lines following incubation with cortisol (Dong et al., 2011). Together, these results suggest that the C allele at rs3027172 may increase risk for stress-associated problematic alcohol use by disrupting affinity of the Snail1 transcription factor with the *PER1* promoter and thereby reducing stress-related *PER1* expression. However, as *PER1* expression and cortisol were not assessed in participants of this study, this interpretation remains speculative.

### *PER1* rs3027172 genotype, early life stress, and reward-related ventral striatum reactivity: the need to account for covariate interactions

A recent review (Keller, 2014) highlights that gene × environment interaction studies have not appropriately controlled for interactions between confounding variables and variables of interest, likely contributing to the low replication rate (27%) of gene × environment findings (Duncan and Keller, 2011). Thus, following these recommendations, terms accounting for potentially confounding ELS and *PER1* genotype interactions with covariates were added to the *PER1* × ELS models predicting problematic alcohol use and ventral striatum reactivity (Keller, 2014). The *PER1* × ELS interaction continued to predict problematic alcohol use even after these additional covariates were added. However, the *PER1* × ELS interaction no longer significantly predicted ventral striatum reactivity after including gene × covariate and environment × covariate interaction terms. *Post-hoc* examination of these analyses revealed that the addition of CTQ × the first ancestral principal component (PC1) and CTQ × PSQI were significantly associated with ventral-striatal reactivity. As PC1 correlates with *PER1* genotype and the CTQxPC1 interaction term correlates with membership to the African American and Asian2 ethnic subgroups, this result may reflect relatively low numbers of minor-allele carriers in these populations (Supplemental Table 4), as well as ethnic subgroup differences in ELS exposure and drinking behavior.

It is intriguing that those with high ELS and poor sleep quality were characterized by relatively blunted VS reactivity to reward (Supplemental Figure 6), as sleep disruption, similar to stress, is also predictive of drug and alcohol problems (Wong et al., 2010). Sleep disruption has been previously associated with blunted striatal activation during a reward task (Holm et al., 2009), and familial risk for alcoholism has been linked to blunted striatal reactivity to reward in young adults (Yau et al., 2012). Thus, this incidental finding would suggest that the blunted ventral striatum reactivity observed may reflect that these participants, who experienced elevated levels of childhood stress and report greater levels of current sleep disruption, are at greater risk for drug and alcohol abuse, and warrants further study.

The present study is not without its limitations. It is first important to consider that participants were university students, and thus results may not be entirely generalizable to the broader population. Epidemiological data suggest that alcohol use is heaviest in young adult years (Fillmore et al., 1991; Naimi et al., 2003) with problematic usage tapering off in the majority of individuals when they reach their mid-20 s (Jackson et al., 2001). Given that more problematic usage in college is predictive of later AUD (Schulenberg et al., 2001), these data identify important factors (i.e., ELS and *PER1* variation) contributing to risk for problematic drinking in college, which in turn, confers risk for post-college AUD. With regard to ELS, CTQ total scores in this sample (i.e., *M* = 33.24) were comparable to other community (e.g., metropolitan Memphis, Tennessee area, *n* = 1007, *M* = 31.7; Scher et al., 2001) and college samples (e.g., UCSD; *n* = 949, *M* = 35.2; Wright et al., 2001), but are considerably lower than those typically observed in clinical samples (e.g., alcohol dependent inpatients *n* = 100, *M* = 42.8; Schäfer et al., 2007), and major depressive disorder and bipolar outpatients *n* = 40, *M* = 47.8 (Watson et al., 2007). These results suggest that the moderating effect of *PER1* variation on problematic drinking arises at ELS levels that are slightly above average (i.e., 35.5, See Johnson-Neyman area of significance in Figure 1). However, it is important to consider that this relatively high functioning college student population may have had other protective factors that may have counteracted the effects of early life adversity.

Second, we must consider the limitations of our phenotypic assessments. With the exception of reward-related ventral striatum reactivity measures and *PER1* variation, all other variables relied upon self-report. It is particularly important to note that the retrospective recall of stress, occurring either recently or early in life, may encompass errors or be influenced by current mood or perception (Monroe, 2008). However, reports have demonstrated that the ELS questionnaire used here and clinician-rated childhood abuse interviews demonstrate convergent validity (Scher et al., 2001). Another consideration is that while our blocked fMRI paradigm increases power to measure VS reactivity, it does so at the cost of some specificity (e.g., separating anticipation of reward from outcome, evaluating reward learning). This is particularly important in light of observations that reward processing is not a monolithic phenomenon and can be dissected into anticipatory, consummatory, and learning components (Berridge et al., 2009). Thus, the finding of no association between the *PER1* × ELS interaction and VS reactivity in the present study does not rule out the possibility that this interaction may be associated with the neurobiological correlates of specific phases of reward processing.

Third, while the *PER1* × ELS interaction predicting problematic alcohol usage was significant in the full sample when accounting for ancestrally informative principal components, it did not reach significance in the majority of our ancestrally homogenous subsamples (Supplemental Table 8). However, consistent with results from the entire sample, each ancestral subsample, showed an interactive effect similar to that observed in the full sample. Our power analysis suggests that subsample analyses were underpowered to detect the association. Notably, this effect did reach statistical significance in the Asian 1 subsample. Future research in various ancestral populations would be informative to clarify whether this association differs according to ancestral origin. It is possible that the findings in the full sample reflect a false positive, despite our best efforts to control for potentially confounding variables (Keller, 2014), a prior report that is consistent with these data (Dong et al., 2011), and rodent work which is consistent with these results (Dong et al., 2011). Given the lack of consistency in many gene × environment interaction studies (Duncan and Keller, 2011) as well as the lack of significance in the European/European American subsample of the present study (the largest subsample), further replication of the reported results is clearly needed.

Fourth, our study did not collect measures of HPA axis function such as cortisol. Given evidence that rs3027172 genotype influences *PER1* expression in the context of cortisol (Dong et al., 2011), it will be important for future research to assess whether ELS-related differences in cortisol mediate relationships between genotype and brain function and behavior. Ideally such investigation would be within the context of longitudinal studies.

Fifth, because this study is cross-sectional, we are unable to firmly establish predictive relationships between *PER1* genotype, ELS, and drinking behavior. That is, although our models imply a direction of effect, we cannot definitively determine if variability in one variable precedes variability in another. In particular, as already noted, the CTQ is retrospective and may be biased by current state. However, given the nature of our measures, a causal relationship is plausible. *PER1* rs3027172 genotype was established prior to the onset of behavior, and our self-report measures assess the occurrence of events that are non-temporally overlapping. The CTQ assesses ELS before the age of 17, and the AUDIT assesses drinking behavior in the past year—all participants are over 18.

These limitations notwithstanding, the results of the present study extend evidence that ELS increases problematic alcohol use in *PER1* rs3027172 minor C allele carriers (Dong et al., 2011). Moreover, the lack of significant ventral striatum results after appropriately controlling for potential interactive confounds, highlights the need for interaction research to properly control for covariates in an effort to reduce false-positive reports (Keller, 2014).

## Author contributions

DB and RB wrote the draft of this manuscript. DAAB, CI, CEC, NSC performed statistical tests. AAP provided expertise on circadian systems. ARH designed the data collection protocol. All authors reviewed, edited, and approved the manuscript.

## Funding

The Duke Neurogenetics Study is supported by Duke University and the National Institutes of Health (NIDA DA033369). ARH receives additional support from the National Institutes of Health (NIDA DA031579). NSC was supported by NIMH (T32-MH014677). YSN was supported by HHMI international student fellowship. DB was supported by NIMH (T32-GM008151) and NSF (DGE-1143954). RB was supported by the Klingenstein Third Generation Foundation and receives additional support from the National Institutes of Health (NIA R01-AG045231). AP received support from the National Heart, Lung, and Blood Institute (K08HL112961). CEC received support from the National Science Foundation (DGE-1143954) and the Mr. and Mrs. Spencer T. Olin Fellowship for Women in Graduate Study.

### Conflict of interest statement

EC works for the commercial entity 23andMe, the company that genotyped the DNS samples through research collaboration (no payment). The other authors declare that the research was conducted in the absence of any commercial or financial relationships that could be construed as a potential conflict of interest.
