# Supplementary material for: PER1 rs3027172 Genotype Interacts with Early Life Stress to Predict Problematic Alcohol Use, but Not Reward-Related Ventral Striatum Activity
Source: Front Psychol. 2016 Mar 31;7:464. doi: 10.3389/fpsyg.2016.00464 (PMC4814479; doi:10.3389/fpsyg.2016.00464)
Supplement: Supplementary file 1 [file DataSheet1.docx]

**Supplemental Table 1.** Effect of exclusion due to non-availability of imaging data on self-report variables, self-report ethnicity, PER1 rs3027172 genotype, and presence of psychiatric diagnosis. *PER1* rs3027172 was unavailable for two participants excluded due to lack of imaging data.

|  | **Included (SD)**  **n=665** | | **Not included (SD)**  **n=62** | **t/x­­^2^** | | ***p*** | |
| --- | --- | --- | --- | --- | --- | --- | --- |
| **CTQ** | 33.06 (7.65) | | 33.36 (8.41) | -0.266 | 0.791 | |  |
| **AUDIT** | 4.84 (3.72) | | 5.01 (4.22) | -0.301 | 0.763 | |  |
| **PSQI** | 4.76 (2.54) | | 4.93 (2.60) | -0.492 | 0.623 | |  |
| **PER1 rs3027172 (carriers) *** | n=182 | | n=17 | 0.006 | 0.939 | |  |
| **Gender (Male)*** | | n=293 | n=26 | 0.104 | 0.747 | |  |
| **Psychiatric Diagnosis*** | | n=52 | n=4 | 0.120 | 0.729 | |  |
| **Caucasian*** | n=305 | | n=27 | 0.123 | 0.726 | |  |
| **African American*** | n=73 | | n=10 | 1.477 | 0.222 | |  |
| **Asian*** | n=187 | | n=14 | 0.870 | 0.351 | |  |
| **Hispanic*** | n=39 | | n=5 | 0.483 | 0.487 | |  |
| **Other*** | n=62 | | n=5 | 0.107 | 0.743 | |  |
| **Age** | 19.47 (1.20) | | 19.64 (1.24) | -1.070 | 0.385 | |  |

CTQ = childhood trauma questionnaire, AUDIT = alcohol use disorders identification test. PSQI = Pittsburgh Sleep Quality Inventory.

***** = analyses were run as a chi-squared test. All others were run as t-tests.

**Supplemental Table 2. Distribution and skewness of self-report variables.**

|  | N | Minimum | Maximum | Mean | Std. Deviation | Skewness | |
| --- | --- | --- | --- | --- | --- | --- | --- |
|  | Statistic | Statistic | Statistic | Statistic | Statistic | Statistic | Std. Error |
| CTQTot | 665 | 25 | 59.0405 | 33.24115 | 7.953584 | 1.282 | 0.095 |
| PSQI | 665 | 0 | 12.8329 | 4.907513 | 2.530686 | 0.739 | 0.095 |
| AUDITTOT | 665 | 0 | 18.0477 | 4.985393 | 4.149198 | 0.862 | 0.095 |

**Supplemental Table 3.** Effect of gender on self-report variables, VS reactivity, presence of psychiatric diagnosis, and ancestral subsamples.

|  | **Men (SD)**  **n=293** | | **Women (SD)**  **n=372** | **t/x­­^2^** | | ***p*** | |
| --- | --- | --- | --- | --- | --- | --- | --- |
| **CTQ** | 33.59 (8.05) | | 32.96 (7.88) | 1.016 | 0.310 | |  |
| **AUDIT** | 6.15 (4.53) | | 4.07 (3.57) | 6.633 | **<0.001** | |  |
| **AUDIT>8*** | n=102 | | n=55 | 36.451 | **<0.001** | |  |
| **PSQI** | 4.77 (2.35) | | 5.02 (2.66) | -1.248 | 0.213 | |  |
| **Bilateral VS Reactivity** | 0.0882 (0.168) | | 0.0588 (0.142) | 2.431 | **0.015** | |  |
| **Psychiatric Diagnosis*** | | n=25 | n=27 | 0.369 | 0.543 | |  |
| **Caucasian*** | n=146 | | n=159 | 3.316 | 0.069 | |  |
| **African American*** | n=17 | | n=56 | 14.356 | **<0.001** | |  |
| **Asian 1*** | n=24 | | n=20 | 2.102 | 0.147 | |  |
| **Asian 2*** | n=60 | | n=83 | 3.27 | 0.568 | |  |
| **Hispanic*** | n=20 | | n=19 | 0.877 | 0.349 | |  |
| **Other*** | n=26 | | n=35 | 0.56 | 0.812 | |  |
| **Age** | 19.69 (1.28) | | 19.6 (1.21) | 0.971 | 0.35 | |  |

CTQ = childhood trauma questionnaire, AUDIT = alcohol use disorders identification test. PSQI = Pittsburgh Sleep Quality Inventory. VS = ventral striatum.

***** = analyses were run as a chi-squared test. All others were run as t-tests.

**Supplemental Table 4.** Effect of Eigenstrat-determined ancestral background on age, self-report variables, VS reactivity, and *PER1* rs3027172 frequency.

|  | **Caucaisan (C)**  **n=305** | **African American (AA) n=73n=73** | **Asian1 (A1)**  **n=44** | **Asian2 (A2)**  **n=143** | **Hispanic (H)**  **n=39** | **Other (O)**  **n=61** | **F/X^2^** | ***p*** |
| --- | --- | --- | --- | --- | --- | --- | --- | --- |
| **CTQ** | 31.09 (6.71) | 37.13 (9.64) | 33.27 (7.64) | 35.99(8.55) | 32.89 (7.52) | 33.07 (7.05) | 12.340 | **<0.001** |
| **AUDIT** | 5.5 (4.21) | 4.27 (4.14) | 4.93 (4.91) | 4.20 (3.56) | 6.26 (4.61) | 4.35 (3.84) | 3.457 | **0.004** |
| **AUDIT>8*** | n=85 | n=13 | n=10 | n=26 | n=14 | n=9 | 12.703 | **0.026** |
| **PSQI** | 4.81 (2.38) | 5.75 (2.59) | 4.36 (2.67) | 4.81 (2.57) | 5.12 (2.97) | 4.88 (2.58) | 2.241 | **0.049** |
| **Bilateral**  **VS Reactivity** | 0.0824 (0.1436) | 0.0650 (0.1516) | 0.0965 (0.1663) | 0.0625 (0.1719) | 0.0147 (0.1545) | 0.0673 (0.1572) | 1.722 | 0.127 |
| **Age** | 19.74 (1.23) | 19.6 (1.13) | 19.14 (1.15) | 19.62 (1.25) | 19.59 (1.33) | 19.69 (1.09) | 1.895 | 0.093 |
| **Psychiatric Diagnosis*** | n=23 | n=9 | n=1 | n=12 | n=3 | n=4 | 4.171 | 0.525 |
| ***PER1 minor C carriers**** | n=117 (MAF=0.44) | n=15  (MAF=0.21) | n=6  (MAF=0.14) | n=14  (MAF=0.10) | n=12  (MAF=0.36) | n=18  (MAF=0.30) | 47.018 | **<0.001** |

**Means are presented with SD indicated in ().**

CTQ**:** Childhood Trauma Questionnaire

AUDIT: Alcohol Use Disorders Identification Test

PSQI: Pittsburgh Sleep Quality Index

Post-hoc t-tests of CTQ scores showed significant differences (ps < .05) for C<AA, C<A2, AA>A1, AA>H, AA>O, A2>A1, A2>H, and A2>O. For AUDIT scores there were significant differences (ps < .05) for C>AA, C>A2, C>O, H>AA, and H>A2. For PSQI scores there were significant differences (ps < .05) for AA>C, AA>A1, AA>A2, AA>O, and O>A1.

Post-hoc comparison of *PER1* minor-allele frequencies showed that the C subsample had more minor-allele carriers than expected, while the A1 and A2 subsamples had fewer minor-allele carriers than expected.

***** = analyses were run as a chi-squared test. All others were run as ANOVAs.

**Supplemental Table 5.** Effect of *PER1* rs3027172 on self-report variables, VS reactivity, presence of psychiatric diagnosis, and ancestral subsamples.

|  | **Major Homozygotes (T/T)**  **n=483** | **Minor-Carriers (C/T & C/C)**  **n=182** | **t/x^2^** | ***p*** |
| --- | --- | --- | --- | --- |
| **CTQ** | 33.49 (7.83) | 32.58 (8.26) | 1.321 | 0.187 |
| **AUDIT** | 4.79 (4.13) | 5.50 (4.16) | -1.970 | **0.049** |
| **AUDIT>8*** | n=106 | n=51 | 2.706 | 0.100 |
| **PSQI** | 4.79 (2.57) | 5.21 (2.40) | -1.871 | 0.062 |
| **Bilateral VS Reactivity** | 0.0659 (0.1579) | 0.0873 (0.1452) | -1.585 | 0.113 |
| **Psychiatric Diagnosis*** | n=33 | n=19 | 2.386 | 0.122 |
| **Caucasian*** | n=188 | n=117 | 34.246 | **<0.001** |
| **African American*** | n=58 | n=15 | 1.919 | 0.166 |
| **Asian 1*** | n=38 | n=6 | 4.470 | **0.035** |
| **Asian 2*** | n=129 | n=14 | 28.318 | **<0.001** |
| **Hispanic*** | n=27 | n=12 | 0.241 | 0.623 |
| **Other*** | n=43 | n=18 | 0.155 | 0.694 |
| **Gender*** | Male n=208 | Male n=85 | 0.710 | 0.399 |
| **Age** | 19.64 (1.27) | 19.65 (1.16) | -1.30 | 0.896 |

Mean values presented with SD indicated in ()

CTQ**:** Childhood Trauma Questionnaire

AUDIT: Alcohol Use Disorders Identification Test

PSQI: Pittsburgh Sleep Quality Index

***** = analyses were run as a chi-squared test. All others were run as t-tests.

**Supplemental Table 6. *Per1* rs30272172 and Early-life adversity significantly interact to predict problematic drinking behavior (AUDIT) even after controlling for gene x covariate and environment x covariate interactions.**

|  |  | Standardized Coefficients | | | |
| --- | --- | --- | --- | --- | --- |
| Model |  | Beta | t | | Sig. |
| 1 | (Constant) | 0.00000 | 0.14 | | 0.888 |
|  | PER1xCTQ | 0.08311 | 2.275 | | 0.023 |
|  | PER1 | 0.02511 | 0.662 | | 0.508 |
|  | CTQ | -0.03906 | -0.983 | | 0.326 |
|  | Sex | -0.45919 | -6.44 | | <0.001 |
|  | Age | 0.00819 | | 2.812 | 0.005 |
|  | PC1 | -0.94253 | -2.054 | | 0.040 |
|  | PC2 | 0.48167 | 2.093 | | 0.037 |
|  | PC3 | 0.38632 | 0.407 | | 0.684 |
|  | PC4 | -0.05730 | -0.596 | | 0.552 |
|  | PC5 | -0.08322 | -0.196 | | 0.845 |
|  | Diagnosis | 0.01705 | 0.569 | | 0.569 |
|  | PSQI | 0.08971 | 6.244 | | <0.001 |
| 2 | (Constant) |  | -0.401 | | 0.689 |
|  | PER1xCTQ | 0.12440 | 2.86 | | 0.004 |
|  | PER1 | 0.03167 | 0.763 | | 0.446 |
|  | CTQ | -0.05656 | -1.378 | | 0.169 |
|  | Sex | -0.44912 | -6.192 | | <0.001 |
|  | Age | 0.02641 | 2.606 | | 0.009 |
|  | PC1 | -0.27623 | -2.45 | | 0.015 |
|  | PC2 | 0.95930 | 1.878 | | 0.061 |
|  | PC3 | 0.17948 | 0.763 | | 0.446 |
|  | PC4 | -0.52154 | -0.541 | | 0.588 |
|  | PC5 | -0.08764 | -0.267 | | 0.789 |
|  | Diagnosis | 0.00535 | 0.36 | | 0.719 |
|  | PSQI | 0.04061 | 5.973 | | <0.001 |
|  | PER1xSex | 0.04154 | 1.12 | | 0.263 |
|  | PER1xAge | 0.02270 | 0.332 | | 0.740 |
|  | PER1xPC1 | -0.67215 | -0.661 | | 0.509 |
|  | PER1xPC2 | -0.02694 | -0.105 | | 0.917 |
|  | PER1xPC3 | 1.15016 | 0.889 | | 0.374 |
|  | PER1xPC4 | -0.04809 | -0.093 | | 0.925 |
|  | PER1xPC5 | -3.13558 | -1.52 | | 0.129 |
|  | PER1xDiagnosis | -0.07169 | -0.695 | | 0.488 |
|  | PER1xPSQI | 0.00197 | 0.555 | | 0.579 |
|  | CTQxSex | 0.23220 | 1.465 | | 0.143 |
|  | CTQxAge | 0.03737 | 1.274 | | 0.203 |
|  | CTQxPC1 | 10.71769 | 2.293 | | 0.022 |
|  | CTQxPC2 | -1.28053 | -0.825 | | 0.410 |
|  | CTQxPC3 | -0.28992 | -0.625 | | 0.532 |
|  | CTQxPC4 | 0.60554 | 0.269 | | 0.788 |
|  | CTQxPC5 | 0.15017 | 0.144 | | 0.885 |
|  | CTQxDiagnosis | 0.27597 | 0.532 | | 0.595 |
|  | CTQxPSQI | 0.00408 | 0.185 | | 0.853 |

**Supplemental Table 7. *Per1* rs30272172 and Early-life adversity significantly interact to predict the likelihood of an AUDIT score over 8, which qualifies as problematic drinking behavior, controlling for gene x covariate and environment x covariate interactions.**

|  |  | Standardized Coefficients | | |
| --- | --- | --- | --- | --- |
| Model |  | Beta | z | Sig. |
| 1 | (Constant) |  | -11.601 | <0.001 |
|  | PER1xCTQ | 0.59076 | 2.128 | 0.033 |
|  | PER1 | 0.20794 | 0.75 | 0.453 |
|  | CTQ | -0.46378 | -1.569 | 0.117 |
|  | Sex | -2.59750 | -5.505 | <0.001 |
|  | Age | 0.05302 | 0.819 | 0.413 |
|  | PC1 | -0.74866 | -0.956 | 0.339 |
|  | PC2 | 6.28589 | 1.555 | 0.120 |
|  | PC3 | 1.03993 | 0.735 | 0.462 |
|  | PC4 | -3.86224 | -0.634 | 0.526 |
|  | PC5 | -2.27352 | -1.112 | 0.266 |
|  | Diagnosis | 0.06259 | 0.702 | 0.483 |
|  | PSQI | 0.19009 | 4.389 | <0.001 |
|  | PER1xSex | 0.00788 | 0.033 | 0.973 |
|  | PER1xAge | -0.18799 | -0.448 | 0.654 |
|  | PER1xPC1 | 0.03211 | 0.005 | 0.996 |
|  | PER1xPC2 | 0.84776 | 0.446 | 0.656 |
|  | PER1xPC3 | 3.28782 | 0.436 | 0.663 |
|  | PER1xPC4 | 1.02101 | 0.333 | 0.739 |
|  | PER1xPC5 | -23.06285 | -1.83 | 0.067 |
|  | PER1xDiagnosis | -0.45885 | -0.728 | 0.467 |
|  | PER1xPSQI | 0.01236 | 0.552 | 0.581 |
|  | CTQxSex | 0.95418 | 0.939 | 0.348 |
|  | CTQxAge | 0.20031 | 1.068 | 0.285 |
|  | CTQxPC1 | 52.17043 | 1.658 | 0.097 |
|  | CTQxPC2 | -16.40813 | -1.443 | 0.149 |
|  | CTQxPC3 | -0.97334 | -0.341 | 0.733 |
|  | CTQxPC4 | 13.86418 | 0.934 | 0.350 |
|  | CTQxPC5 | 3.83533 | 0.539 | 0.590 |
|  | CTQxDiagnosis | 3.11261 | 1.019 | 0.308 |
|  | CTQxPSQI | 0.02583 | 0.185 | 0.853 |

**Supplemental Table 8. The interaction of *Per1* rs30272172 and Early-life adversity predicting problematic drinking behavior (AUDIT) in each of the six ethnic subsamples.**

|  | Caucasian | | | African American | | | Asian 1 | | | |
| --- | --- | --- | --- | --- | --- | --- | --- | --- | --- | --- |
|  | Standardized Coefficients | | | Standardized Coefficients | | | Standardized Coefficients | | | |
|  | Beta | t | Sig. | Beta | t | Sig. | Beta | t |  | Sig. |
| (Constant) |  | 0.219 | 0.827 |  | 0.103 | 0.918 |  | 1.011 | 0.3201 | |
| PER1xCTQ | 0.0146 | 0.238 | 0.812 | 0.2195 | 1.691 | 0.096 | 0.6178 | 3.456 | 0.0017 | |
| PER1 | -0.0047 | -0.086 | 0.932 | 0.0770 | 0.686 | 0.496 | 0.0165 | 0.134 | 0.8943 | |
| CTQ | -0.0409 | -0.714 | 0.476 | -0.0917 | -0.819 | 0.416 | -0.3424 | -2.151 | 0.0397 | |
| Sex | -0.6471 | -4.296 | <0.001 | -1.6955 | -4.581 | <0.001 | -0.4672 | -1.068 | 0.2939 | |
| Age | 0.0381 | 1.716 | 0.087 | 0.1216 | 2.235 | 0.029 | -0.0283 | -0.625 | 0.5364 | |
| Diagnosis | -0.0006 | -0.031 | 0.976 | -0.0408 | -0.871 | 0.387 | 0.1510 | 1.048 | 0.3032 | |
| PSQI | 0.0473 | 3.997 | <0.001 | 0.0400 | 2.146 | 0.036 | 0.0564 | 3.029 | 0.005 | |
| PER1xSex | 0.0109 | 0.250 | 0.803 | 0.1255 | 0.877 | 0.384 | 0.7021 | 2.761 | 0.0097 | |
| PER1xAge | 0.0336 | 0.258 | 0.797 | 0.2633 | 0.629 | 0.532 | 1.2691 | 2.281 | 0.0298 | |
| PER1xDiagnosis | -0.0487 | -0.236 | 0.813 | -0.2486 | -0.277 | 0.783 | NA | NA | NA | |
| PER1xPSQI | -0.0034 | -0.664 | 0.507 | 0.0162 | 1.056 | 0.295 | -0.1093 | -1.650 | 0.1094 | |
| CTQxSex | 0.2892 | 0.976 | 0.330 | 1.3655 | 2.632 | 0.011 | 0.0925 | 0.657 | 0.5161 | |
| CTQxAge | 0.0525 | 1.058 | 0.291 | -0.0059 | -0.063 | 0.950 | -0.0589 | -0.255 | 0.8004 | |
| CTQxDiagnosis | 2.5393 | 1.673 | 0.096 | 1.4537 | 0.675 | 0.503 | NA | NA | NA | |
| CTQxPSQI | -0.0047 | -0.076 | 0.940 | 0.0344 | 0.362 | 0.719 | -0.1976 | -3.302 | 0.0025 | |
|  | Asian 2 | | | Hispanic | | | Other | | | |
|  | Standardized Coefficients | | | Standardized Coefficients | | | Standardized Coefficients | | | |
|  | Beta | t | Sig. | Beta | t | Sig. | Beta | t |  | Sig. |
| (Constant) |  | 0.095 | 0.925 |  | 0.175 | 0.862 |  | 0.347 | 0.7302 | |
| PER1xCTQ | 0.1523 | 1.873 | 0.063 | -0.0222 | -0.108 | 0.915 | 0.1000 | 0.713 | 0.4797 | |
| PER1 | -0.0127 | -0.139 | 0.890 | 0.6533 | 0.980 | 0.337 | 0.0832 | 0.545 | 0.5881 | |
| CTQ | 0.1450 | 1.664 | 0.099 | -0.2743 | -0.499 | 0.623 | -0.1926 | -1.141 | 0.26 | |
| Sex | -0.3372 | -1.394 | 0.166 | -0.0112 | -0.022 | 0.983 | -0.8170 | -1.954 | 0.0569 | |
| Age | 0.0505 | 2.104 | 0.037 | 0.1068 | 1.338 | 0.194 | -0.0460 | -0.651 | 0.5185 | |
| Diagnosis | 0.0428 | 2.085 | 0.039 | -0.1103 | -0.160 | 0.874 | -0.1178 | -1.683 | 0.0993 | |
| PSQI | 0.0252 | 2.494 | 0.014 | 0.0843 | 3.012 | 0.006 | -0.0175 | -0.641 | 0.5248 | |
| PER1xSex | 0.1679 | 1.105 | 0.271 | 0.0009 | 0.006 | 0.995 | -0.1219 | -0.821 | 0.4161 | |
| PER1xAge | 0.1753 | 0.383 | 0.702 | -0.4935 | -1.149 | 0.262 | -0.1824 | -0.304 | 0.7622 | |
| PER1xDiagnosis | -0.3491 | -0.719 | 0.474 | -0.8346 | -0.064 | 0.950 | -0.0298 | -0.051 | 0.9599 | |
| PER1xPSQI | 0.0062 | 0.773 | 0.441 | 0.0525 | 3.164 | 0.004 | -0.0083 | -0.653 | 0.517 | |
| CTQxSex | 0.0151 | 0.071 | 0.943 | -0.5481 | -0.751 | 0.460 | 1.3979 | 2.135 | 0.0383 | |
| CTQxAge | -0.0661 | -1.120 | 0.265 | -0.3051 | -2.187 | 0.039 | 0.2007 | 1.879 | 0.0667 | |
| CTQxDiagnosis | -4.3074 | -2.478 | 0.015 | 6.3800 | 0.129 | 0.898 | -3.4493 | -0.427 | 0.6717 | |
| CTQxPSQI | 0.1233 | 2.443 | 0.016 | -0.5383 | -2.394 | 0.025 | -0.0586 | -0.448 | 0.6567 | |

**Supplemental Table 9. *Per1* rs30272172 and Early-life adversity significantly interact to predict problematic drinking behavior (AUDIT) when including participants originally excluded due to lack of neuroimaging data (n=719)**

|  |  | Standardized Coefficients | | |
| --- | --- | --- | --- | --- |
| Model |  | Beta | t | Sig. |
| 1 |  | Estimate | t | p |
|  | (Constant) | 0.00000 | -0.481 | 0.631 |
|  | PER1xCTQ | 0.12899 | 3.18 | 0.002 |
|  | PER1 | 0.02569 | 0.64 | 0.522 |
|  | CTQ | -0.06265 | -1.588 | 0.113 |
|  | Sex | -0.24119 | -6.742 | <0.001 |
|  | Age | 0.07908 | 2.224 | 0.027 |
|  | PC1 | -0.11301 | -2.763 | 0.006 |
|  | PC2 | 0.08501 | 2.181 | 0.030 |
|  | PC3 | 0.03582 | 0.929 | 0.353 |
|  | PC4 | -0.01396 | -0.394 | 0.694 |
|  | PC5 | -0.00892 | -0.253 | 0.801 |
|  | PSQI | 0.24666 | 6.687 | <0.001 |
|  | Diagnosis | 0.01260 | 0.322 | 0.748 |
|  | PER1xSex | 0.03121 | 0.872 | 0.383 |
|  | PER1xAge | 0.00386 | 0.103 | 0.918 |
|  | PER1xPC1 | -0.02551 | -0.518 | 0.605 |
|  | PER1xPC2 | 0.01055 | 0.277 | 0.782 |
|  | PER1xPC3 | 0.04689 | 1.001 | 0.317 |
|  | PER1xPC4 | -0.00723 | -0.191 | 0.848 |
|  | PER1xPC5 | -0.05228 | -1.491 | 0.136 |
|  | PER1xPSQI | 0.03204 | 0.822 | 0.412 |
|  | PER1xDiagnosis | -0.03303 | -0.958 | 0.338 |
|  | CTQxSex | 0.05196 | 1.411 | 0.159 |
|  | CTQxAge | 0.04450 | 1.272 | 0.204 |
|  | CTQxPC1 | 0.09390 | 2.41 | 0.016 |
|  | CTQxPC2 | -0.03406 | -1.005 | 0.315 |
|  | CTQxPC3 | -0.02481 | -0.671 | 0.503 |
|  | CTQxPC4 | 0.00100 | 0.028 | 0.978 |
|  | CTQxPC5 | 0.01056 | 0.284 | 0.777 |
|  | CTQxPSQI | 0.00569 | 0.169 | 0.866 |

**Supplemental Table 10. *Per1* rs3027172 and Early-life adversity do not significantly interact to predict ventral striatal reactivity when controlling for gene x covariate and environment x covariate interactions.**

|  |  | Standardized Coefficients | | |
| --- | --- | --- | --- | --- |
| Model |  | Beta | t | Sig. |
| 1 | (Constant) |  | 0.132 | 0.895 |
|  | PER1xCTQ | 0.08380 | 2.145 | 0.032 |
|  | PER1 | 0.06695 | 1.651 | 0.099 |
|  | CTQ | -0.04072 | -0.958 | 0.338 |
|  | Sex | -0.17220 | -2.258 | 0.024 |
|  | Age | -0.00505 | -1.62 | 0.106 |
|  | PC1 | -0.00742 | -0.015 | 0.988 |
|  | PC2 | 0.01349 | 0.055 | 0.956 |
|  | PC3 | 0.63155 | 0.622 | 0.534 |
|  | PC4 | 0.07347 | 0.714 | 0.475 |
|  | PC5 | 0.30387 | 0.669 | 0.504 |
|  | Diagnosis | 0.01000 | 0.312 | 0.755 |
|  | PSQI | -0.01828 | -1.19 | 0.235 |
| 2 | (Constant) |  | 1.315 | 0.189 |
|  | PER1xCTQ | 0.05643 | 1.231 | 0.219 |
|  | PER1 | 0.06268 | 1.434 | 0.152 |
|  | CTQ | -0.01241 | -0.287 | 0.774 |
|  | Sex | -0.18633 | -2.437 | 0.015 |
|  | Age | -0.01375 | -1.287 | 0.199 |
|  | PC1 | 0.05786 | 0.487 | 0.627 |
|  | PC2 | -0.03814 | -0.071 | 0.944 |
|  | PC3 | 0.10406 | 0.42 | 0.675 |
|  | PC4 | 1.13770 | 1.12 | 0.263 |
|  | PC5 | 0.38340 | 1.107 | 0.269 |
|  | Diagnosis | 0.01820 | 1.16 | 0.246 |
|  | PSQI | -0.00526 | -0.734 | 0.463 |
|  | PER1xSex | 0.06082 | 1.556 | 0.120 |
|  | PER1xAge | -0.02018 | -0.28 | 0.780 |
|  | PER1xPC1 | -0.46548 | -0.434 | 0.664 |
|  | PER1xPC2 | -0.29333 | -1.082 | 0.280 |
|  | PER1xPC3 | -0.73396 | -0.538 | 0.590 |
|  | PER1xPC4 | -0.32851 | -0.605 | 0.545 |
|  | PER1xPC5 | -1.67776 | -0.772 | 0.441 |
|  | PER1xDiagnosis | -0.16886 | -1.553 | 0.121 |
|  | PER1xPSQI | 0.00510 | 1.364 | 0.173 |
|  | CTQxSex | 0.27827 | 1.666 | 0.096 |
|  | CTQxAge | -0.00593 | -0.192 | 0.848 |
|  | CTQxPC1 | -12.36640 | -2.51 | 0.012 |
|  | CTQxPC2 | 0.20059 | 0.123 | 0.902 |
|  | CTQxPC3 | 0.59427 | 1.215 | 0.225 |
|  | CTQxPC4 | -0.99835 | -0.421 | 0.674 |
|  | CTQxPC5 | -1.87894 | -1.714 | 0.087 |
|  | CTQxDiagnosis | -0.45213 | -0.826 | 0.409 |
|  | CTQxPSQI | -0.07234 | -3.114 | 0.002 |

**Supplemental Table 11. The interaction of *Per1* rs30272172 and Early-life adversity predicting ventral striatal reactivity in each of the six ethnic subsamples.**

|  | Caucasian | | | African American | | | Asian 1 | | |
| --- | --- | --- | --- | --- | --- | --- | --- | --- | --- |
|  | Standardized Coefficients | | | Standardized Coefficients | | | Standardized Coefficients | | |
|  | Beta | t | Sig. | Beta | t | Sig. | Beta | t | Sig. |
| (Constant) |  | 0.501 | 0.617 |  | 0.184 | 0.855 |  | 0.294 | 0.771 |
| PER1xCTQ | 0.08772 | 1.351 | 0.178 | -0.31384 | -2.082 | 0.042 | 0.21491 | 0.808 | 0.426 |
| PER1 | 0.00996 | 0.17 | 0.865 | 0.12838 | 0.985 | 0.329 | 0.02570 | 0.14 | 0.889 |
| CTQ | 0.06633 | 1.093 | 0.275 | -0.00964 | -0.074 | 0.941 | 0.08035 | 0.339 | 0.737 |
| Sex | -0.26175 | -1.642 | 0.102 | -0.94633 | -2.202 | 0.032 | -0.14133 | -0.217 | 0.830 |
| Age | -0.01687 | -0.718 | 0.473 | -0.08550 | -1.353 | 0.181 | 0.02846 | 0.423 | 0.676 |
| Diagnosis | 0.02733 | 1.238 | 0.217 | 0.07216 | 1.327 | 0.190 | -0.07073 | -0.329 | 0.744 |
| PSQI | -0.00625 | -0.499 | 0.618 | -0.02841 | -1.314 | 0.194 | -0.00181 | -0.065 | 0.948 |
| PER1xSex | 0.02901 | 0.627 | 0.531 | -0.02871 | -0.173 | 0.864 | -0.19436 | -0.513 | 0.612 |
| PER1xAge | 0.07696 | 0.559 | 0.576 | 0.16653 | 0.343 | 0.733 | -0.58885 | -0.711 | 0.483 |
| PER1xDiagnosis | -0.24471 | -1.122 | 0.263 | 0.68997 | 0.661 | 0.511 | NA | NA | NA |
| PER1xPSQI | -0.00108 | -0.202 | 0.840 | 0.00491 | 0.275 | 0.784 | 0.09226 | 0.935 | 0.357 |
| CTQxSex | 0.61757 | 1.97 | 0.050 | -0.64824 | -1.076 | 0.286 | 0.08910 | 0.425 | 0.674 |
| CTQxAge | 0.04816 | 0.917 | 0.360 | -0.08222 | -0.753 | 0.454 | 0.15804 | 0.46 | 0.649 |
| CTQxDiagnosis | -1.16741 | -0.727 | 0.468 | -0.82865 | -0.331 | 0.742 | NA | NA | NA |
| CTQxPSQI | -0.09890 | -1.506 | 0.133 | -0.13670 | -1.24 | 0.220 | -0.07186 | -0.807 | 0.426 |
|  | Asian 2 | | | Hispanic | | | Other | | |
|  | Standardized Coefficients | | | Standardized Coefficients | | | Standardized Coefficients | | |
|  | Beta | t | Sig. | Beta | t | Sig. | Beta | t | Sig. |
| (Constant) |  | 0.538 | 0.592 |  | 1.244 | 0.226 |  | 0.019 | 0.985 |
| PER1xCTQ | 0.01329 | 0.156 | 0.876 | 0.25508 | 0.908 | 0.373 | 0.16067 | 1.105 | 0.275 |
| PER1 | 0.00206 | 0.021 | 0.983 | -0.99849 | -1.091 | 0.287 | 0.03870 | 0.245 | 0.808 |
| CTQ | -0.18459 | -2.027 | 0.045 | -0.86761 | -1.149 | 0.263 | 0.13523 | 0.773 | 0.444 |
| Sex | -0.50103 | -1.981 | 0.050 | 0.08126 | 0.114 | 0.910 | 0.47581 | 1.098 | 0.278 |
| Age | -0.00034 | -0.013 | 0.989 | -0.15828 | -1.445 | 0.162 | -0.06020 | -0.822 | 0.415 |
| Diagnosis | -0.01393 | -0.649 | 0.517 | 1.11597 | 1.183 | 0.249 | 0.07887 | 1.087 | 0.283 |
| PSQI | -0.00495 | -0.469 | 0.640 | 0.01784 | 0.464 | 0.647 | -0.01529 | -0.541 | 0.591 |
| PER1xSex | -0.10256 | -0.646 | 0.520 | 0.20057 | 1.055 | 0.302 | 0.30156 | 1.959 | 0.056 |
| PER1xAge | -0.39560 | -0.828 | 0.409 | 0.37238 | 0.632 | 0.534 | -1.03469 | -1.666 | 0.103 |
| PER1xDiagnosis | -0.52694 | -1.038 | 0.301 | -23.38020 | -1.306 | 0.204 | -0.21398 | -0.351 | 0.728 |
| PER1xPSQI | 0.01587 | 1.884 | 0.062 | 0.01315 | 0.577 | 0.570 | -0.00374 | -0.285 | 0.777 |
| CTQxSex | 0.37678 | 1.702 | 0.091 | -0.37181 | -0.371 | 0.714 | 0.15417 | 0.227 | 0.821 |
| CTQxAge | -0.11857 | -1.921 | 0.057 | -0.04776 | -0.249 | 0.805 | 0.07997 | 0.722 | 0.474 |
| CTQxDiagnosis | 0.12147 | 0.067 | 0.947 | -89.10560 | -1.316 | 0.201 | 4.74235 | 0.566 | 0.574 |
| CTQxPSQI | -0.04378 | -0.83 | 0.408 | -0.42551 | -1.378 | 0.181 | -0.15048 | -1.109 | 0.273 |

**Supplemental Figure 1. Ancestral principal components.**


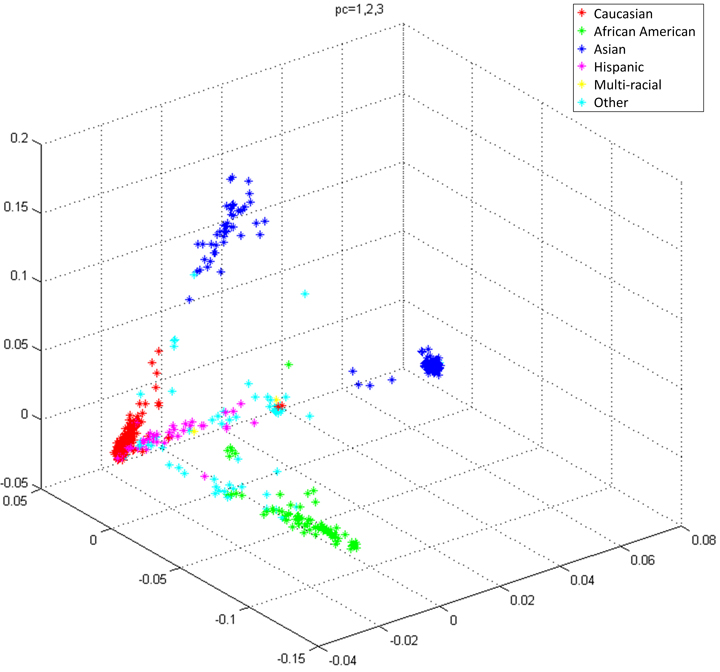


Ancestral principal components 1 – 3 generated using Eigenstrat. Color coding is of self-report of ethnicity. Based on these results participants who self-report as ‘Asian’ were split into two subgroups (Asian1 and Asian2) as their ancestral principle components separated into two distinct clusters according to k means clustering.

**Supplemental Figure 2.** Ventral striatal activation from the Positive>Negative feedback contrast of the Corticostriatal Reactivity task.

14

12

10

8

6

4

2

0


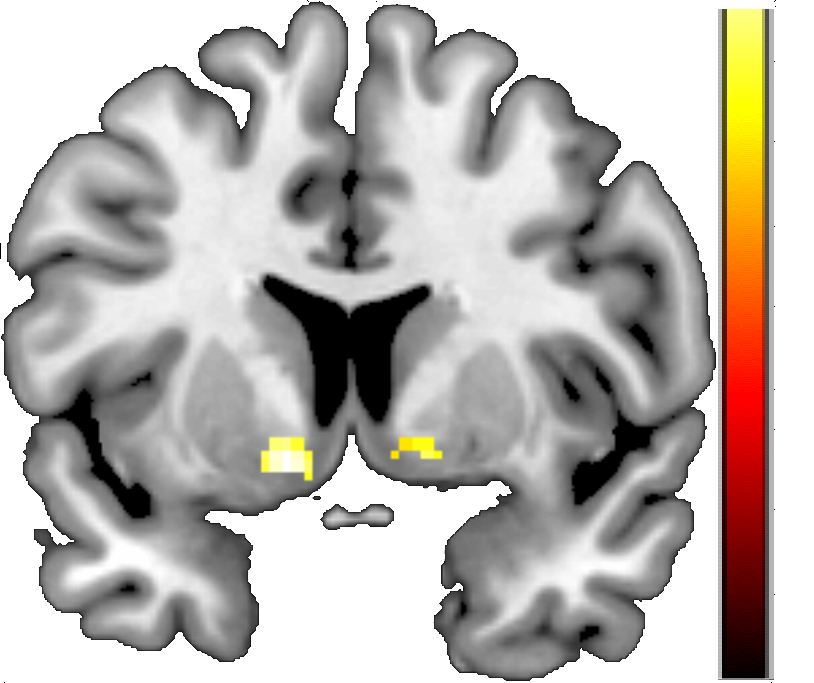


Statistical parametric map illustrating bilateral VS activation clusters for the contrast “positive reward> negative loss” with bilateral spherical 5mm ROIs centered on the points of peak activation from Hariri et al. (2006), overlaid onto a canonical structural brain image Montreal Neurological Institute coordinates and statistics *(p*<.05, family-wise error whole-brain corrected and ≥10 contiguous voxels): left hemisphere: x= -12, y= 8, z= -10, *t* = 13.59, *P*<.001, right hemisphere: x = 12, y = 10, z= -8, t=12.63, *p<.001*.

**Supplemental Figure 3. The interaction of *Per1* rs30272172 and Early-life adversity predicting problematic drinking behavior (AUDIT) in each of the six ethnic subsamples.
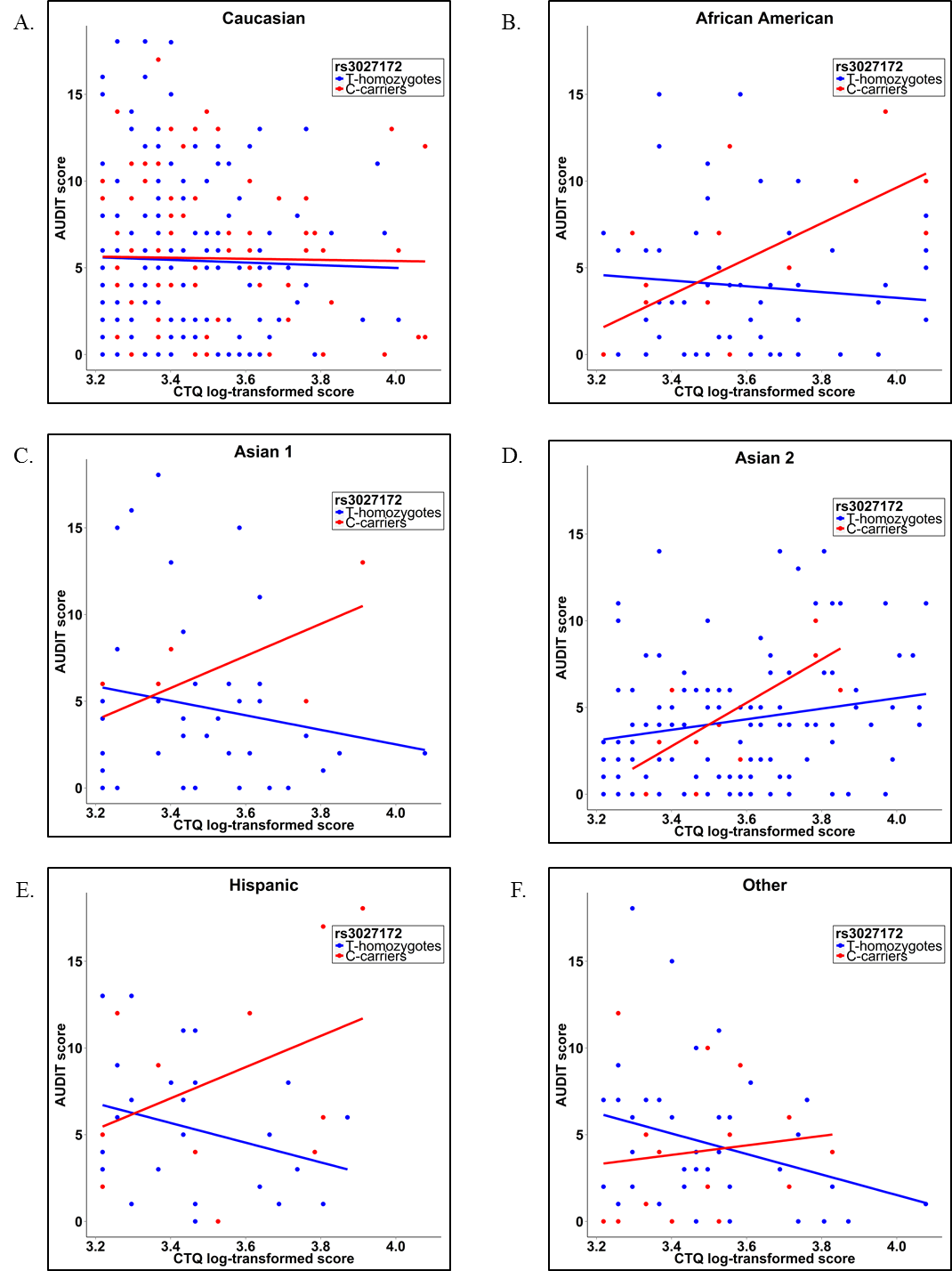
**

**Supplemental Figure 4. *PER1* rs3027172 and Early-Life Adversity Do Not Significantly Interact to Predict VS reactivity.** (ΔR^2^=.0021, b=0.056, t=1.231, p=0.219).

**Supplemental Figure 5. Ancestral Principal Component 1 and Early-Life Adversity Interact to Predict VS reactivity.** (ΔR^2^=.0133, b=-0.132, t=-3.014, p=0.003).

The interaction of the ancestral principal component 1 (PC1) and CTQ scores was found to significantly predict ventral striatal (VS) reactivity when included as a covariate in the analysis examining the interaction of *PER1* rs3027172 and CTQ scores predicting VS reactivity (see Results; b=-12.3664, t=-2.51, p=0.01231). Follow-up analyses examining the interaction of PC1 and CTQ scores on VS reactivity, with age, sex, the other four ancestral principal components, PSQI, and presence of a psychiatric diagnosis, and interactions between these variables and variables of interest (PC1 and CTQ scores) as covariates, found that the PC1xCTQ interaction remained significant (ΔR^2^=.0133, b=-0.132, t=-3.014, p=0.003). It was found that there was a significant negative relationship between CTQ scores and VS reactivity among participants with higher PC1 values (Johnson-Neyman significance for PC1 values greater than 0.0275). Participants were split into three PC1 groups based on the group standard deviation (low= -0.028 - -0.037, medium = -0.038 - 0.037, high = 0.038 - 0.0704). Examination of simple-slopes revealed a significant negative relationship between CTQ-scores and VS reactivity in the high PC1 group of participants (b=-0.173, t=-2.594, p=0.010). This group of high PC1 participants consists predominantly of members of the Asian2 subgroup (see Fig. S1).

**Supplemental Figure 6. Sleep Quality and Early-Life Adversity Interact to Predict VS reactivity.** (ΔR2=.0107, b=-0.1211, t=-2.682, p=0.007).

The interaction of PSQI scores (sleep quality) and CTQ scores was found to significantly predict ventral striatal (VS) reactivity when included as a covariate in the analysis examining the interaction of PER1 rs3027172 and CTQ scores predicting VS reactivity (see Supplemental Table 6; b=-0.0723, t=-3.114, p=0.0019). Follow-up analyses examining the interaction of PSQI and CTQ scores on VS reactivity, with age, sex, the five ancestral principal components, and presence of a psychiatric diagnosis, and interactions between these variables and variables of interest (PSQI and CTQ scores) as covariates, found that the PSQIxCTQ interaction remained significant (ΔR2=.0107, b=-0.1211, t=-2.682, p=0.007). It was found that there was a significant negative relationship between CTQ scores and VS reactivity among participants with higher PSQI values (Johnson-Neyman significance for PSQI scores greater than 7.3). Participants were split into three PSQI groups based on the group standard deviation (low= 0 – 4, medium = 5 - 6, high = 7 - 12). Examination of simple-slopes revealed a significant negative relationship between CTQ-scores and VS reactivity in the high PSQI group of participants (b=-0.176, t=-2.592, p=0.012).
